# Supplementary material for: BLAST-based validation of metagenomic sequence assignments
Source: PeerJ. 2018 May 28;6:e4892. doi: 10.7717/peerj.4892 (PMC5978398; doi:10.7717/peerj.4892)
Supplement: Supplemental Information 1 [file peerj-06-4892-s001.pdf]

BLAST-based validation of metagenomic sequence assignments

Adam L. Bazinet, Brian D. Ondov, Daniel D. Sommer, and Shashikala Ratnayake

Supplementary Information

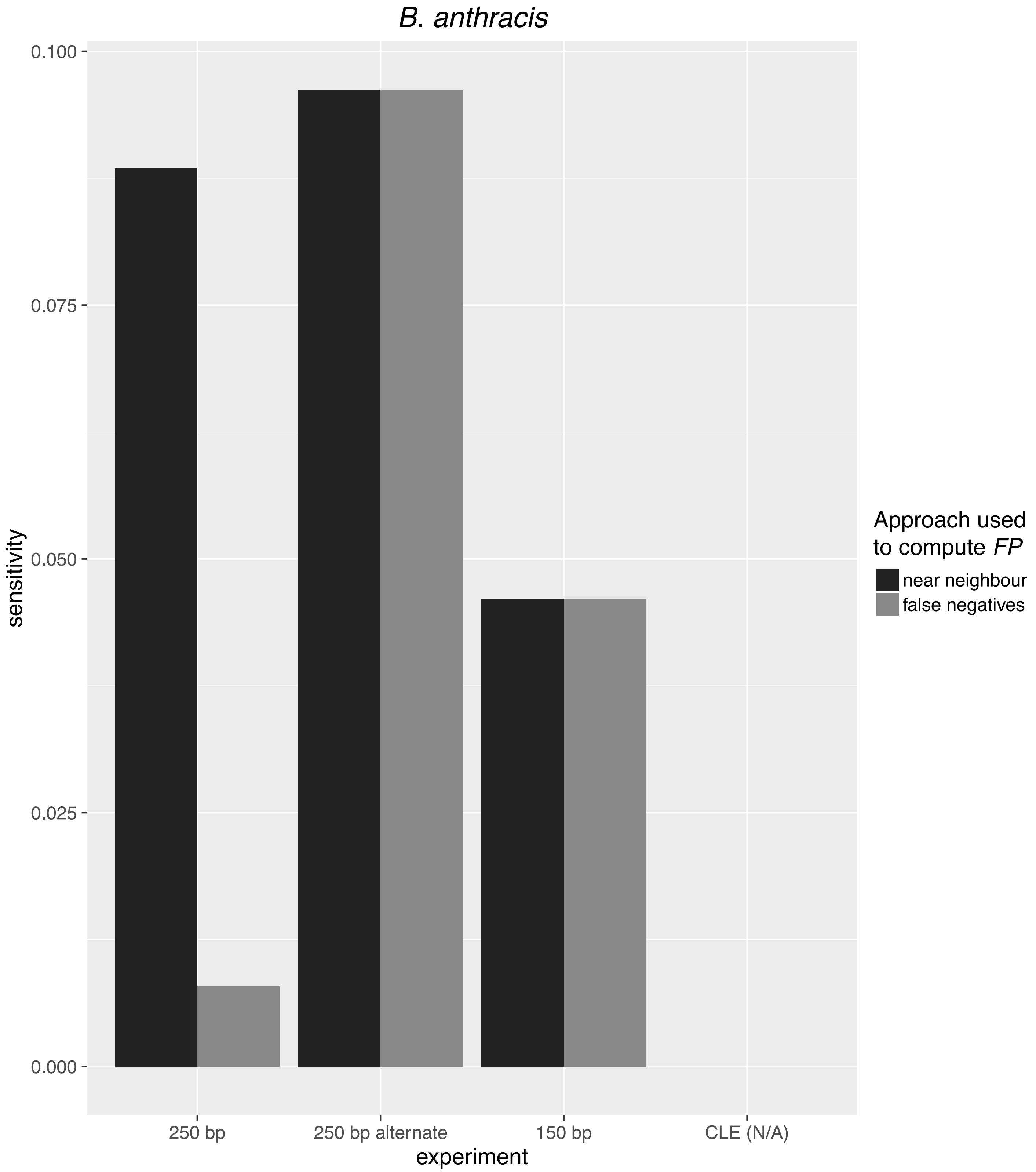

Supplementary Figure S1. Sensitivity for detection of *B. anthracis* is plotted across four experiments: 250-bp simulated reads, 250-bp simulated reads using an alternate representative genome, 150-bp simulated reads, and clade-level exclusion (CLE). The two approaches used to compute false positives, "near neighbour" and "false negatives", are also compared.

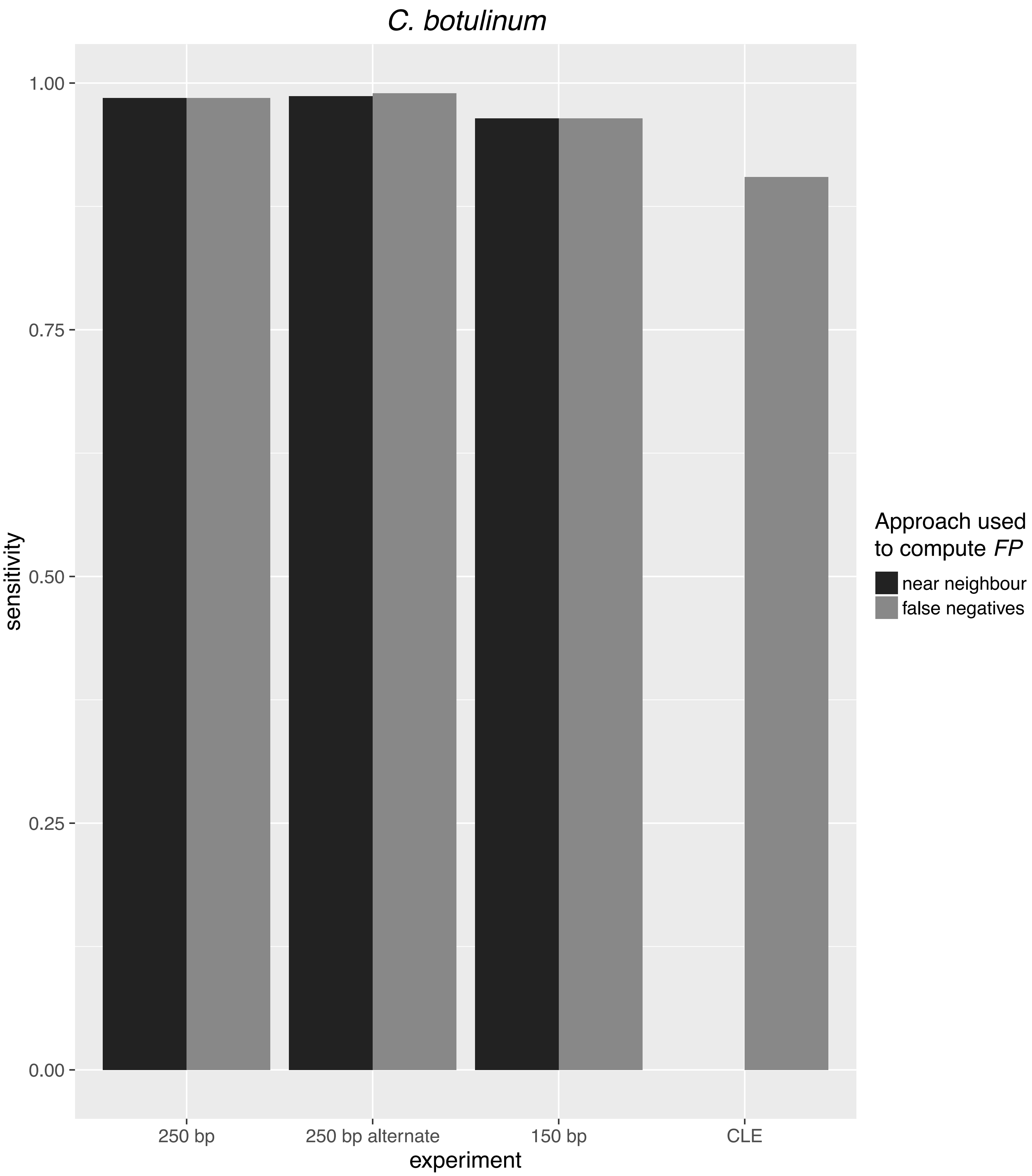

Supplementary Figure S2. Sensitivity for detection of *C. botulinum* is plotted across four experiments: 250-bp simulated reads, 250-bp simulated reads using an alternate representative genome, 150-bp simulated reads, and clade-level exclusion (CLE). The two approaches used to compute false positives, "near neighbour" and "false negatives", are also compared.

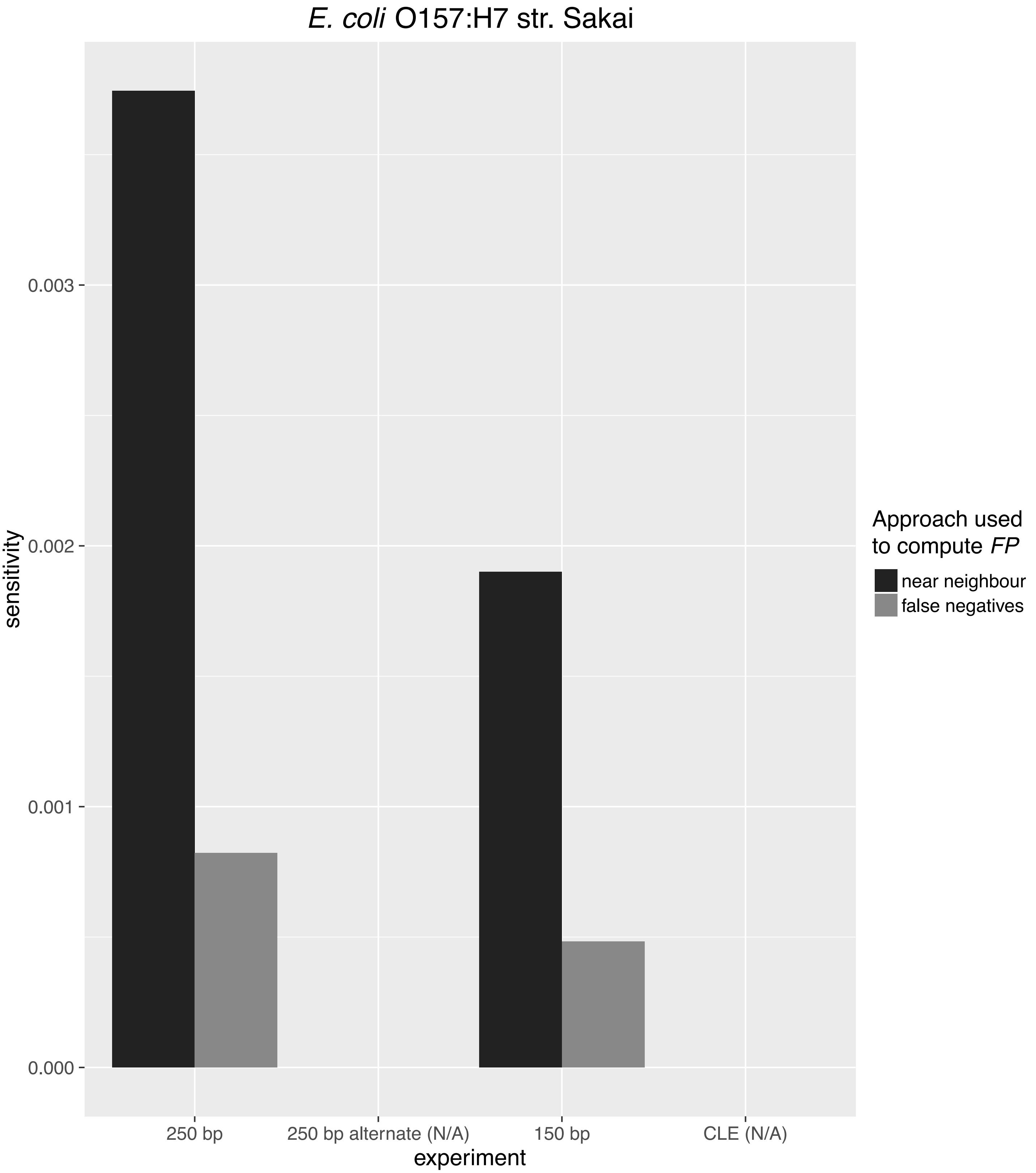

Supplementary Figure S3. Sensitivity for detection of *E. coli* O157:H7 str. Sakai is plotted across four experiments: 250-bp simulated reads, 250-bp simulated reads using an alternate representative genome, 150-bp simulated reads, and clade-level exclusion (CLE). The two approaches used to compute false positives, "near neighbour" and "false negatives", are also compared.

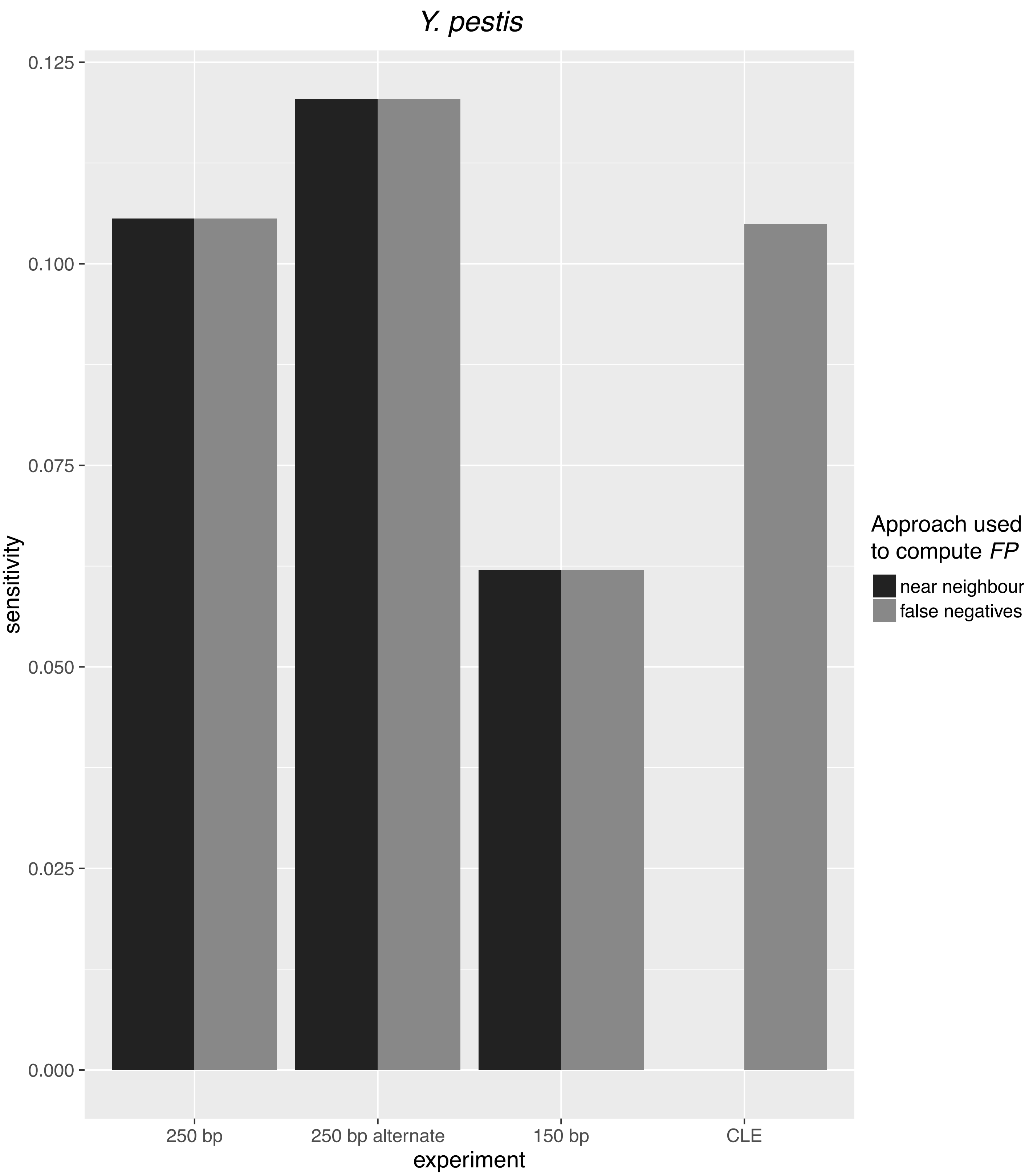

Supplementary Figure S4. Sensitivity for detection of *Y. pestis* is plotted across four experiments: 250-bp simulated reads, 250-bp simulated reads using an alternate representative genome, 150-bp simulated reads, and clade-level exclusion (CLE). The two approaches used to compute false positives, "near neighbour" and "false negatives", are also compared.
